# Longitudinal changes in global and domain specific cognitive function in the very‐old: findings from the Newcastle 85+ Study

**DOI:** 10.1002/gps.4743

**Published:** 2017-06-22

**Authors:** Blossom C. M. Stephan, Graciela Muniz‐Terrera, Antoneta Granic, Joanna Collerton, Karen Davies, Brian K. Saxby, Keith A. Wesnes, Thomas B.L. Kirkwood, Carol Jagger

**Affiliations:** ^1^ Institute of Health and Society Newcastle University, Campus for Ageing and Vitality Newcastle upon Tyne UK; ^2^ Centre for Dementia Prevention University of Edinburgh, Royal Edinburgh Hospital Edinburgh UK; ^3^ AGE Research Group, Institute of Neuroscience Newcastle University, Campus for Ageing and Vitality Newcastle upon Tyne UK; ^4^ Institute for Ageing Newcastle University, Campus for Ageing and Vitality Newcastle upon Tyne UK; ^5^ Department of Psychology Northumbria University Newcastle upon Tyne UK; ^6^ Wesnes Cognition Limited Streatley‐on‐Thames UK; ^7^ Institute for Cell and Molecular Biosciences and Institute for Ageing Newcastle University Newcastle upon Tyne UK; ^8^ NIHR Newcastle Biomedical Research Centre, and Newcastle upon Tyne Hospitals NHS Foundation Trust Newcastle upon Tyne UK

**Keywords:** cognition, very‐old, cognitive trajectories, memory, speed, attention, epidemiology

## Abstract

**Objective:**

Ageing is associated with changes in cognition in some, but not all domains. In young–old adults, defined as persons aged 65–84 years, baseline cognitive function has been shown to impact on cognitive trajectories. Whether similar patterns occur in the very‐old, defined as persons aged 85 years and over, is not known.

**Methods:**

Longitudinal changes (5 years' follow‐up) in global and domain specific cognitive function including memory, attention and speed were investigated in participants from the Newcastle 85+ Study (*n* = 845). At baseline, participants were grouped using Mini‐Mental State Examination cut‐off scores and dementia status into the following: not impaired, mildly impaired or severely impaired/dementia groups.

**Results:**

Only a limited number of cognitive measures showed significant decline in performance over time. Where observed, change generally occurred only in the severely impaired group. In the severely impaired group, small differences in baseline age were associated with poorer performance over time on most measures. Education was not protective against cognitive decline in any group.

**Conclusions:**

There are individuals who maintain a high level of cognitive function or only show mild impairments even into their ninth decade of life. This group of successful cognitive agers may provide insight for identifying predictors of cognitive integrity in later life. In individuals with severe impairment, cognitive performance shows significant decline over time, especially in measures of attention and speed. Further work to identify those individuals at highest risk of cognitive decline is necessary to implement early support and intervention strategies in this rapidly expanding age group. © 2017 The Authors. *International Journal of Geriatric Psychiatry* published by John Wiley & Sons Ltd.

## Introduction

Longitudinal studies have reported large individual variability in cognitive performance in older aged adults with some individuals showing a decline in functioning, others remaining stable and some even improving (Brayne *et al.,* 1992; Lyketsos *et al.,* 1999; Howieson *et al.,* 2003; Terrera *et al.,* 2010). Individual differences may be explained by variability in resilience to the effects of ageing through, for example, reserve or compensation, health‐related factors or learning effects. Cognitive decline has been associated with an increased risk of mild cognitive impairment and dementia and can impede independence and mobility. Therefore, in older populations, knowing which cognitive domains change as a function of time (versus disease processes), and the measures that are most sensitive to detect and monitor change over time are important not only for creating diagnostic protocols but also for the development and testing of personalised interventions focused on improving or maintaining cognitive function in later life.

Exactly which cognitive domains are and are not resilient to ageing has been extensively studied. Generally, although not always, general knowledge improves with age, while speed, attention, inhibition and memory decline, and abstraction has been found to remain stable (Goh *et al.,* 2012). However, whether these trends are also observed in the very‐old, defined as persons aged 85 years and older, is not known. Indeed, studies investigating trajectories of cognitive function in older populations have largely been derived from samples of young–old persons (e.g. 65–84 years) or samples with very broad age ranges (e.g. from mid to later life). As such, they may not be representative of the very‐old population who are a unique survivor cohort. Further, the very‐old are the most rapidly growing age segment of the population and have the highest risk of cognitive decline and dementia (Kinsella and He, 2009). Individuals aged 85 years and over may have greater sensory (e.g. vision and hearing) and physical impairment, and comorbidities that may influence cognitive performance (Collerton *et al.,* 2009; Jefferis *et al.,* 2013). They may also form a select cohort characterised by a more protective biological profile and have better health or lifestyles that increase resilience. Each of these factors could modify cognitive trajectories in this age group (Ankri and Poupard, 2003; Slavin *et al.,* 2013). Thus, while research suggests that cognitive impairment is common in very‐old age, it may not necessarily be inevitable (Kliegel *et al.,* 2004).

In this study, we investigated, for the first time, cognitive trajectories of global and domain specific functioning including memory, attention and speed, in a cognitively diverse sample of individuals aged 85 years at baseline. As baseline functioning has been found to impact cognitive trajectories in young–old samples (de Frias *et al.,* 2009; Mungas *et al.,* 2010), we investigated longitudinal changes in groups defined using baseline Mini‐Mental State Examination (MMSE) cut‐off scores and dementia status with individuals classified into the following: not impaired (MMSE 26–30), mildly impaired (MMSE 22–25) or severely impaired/dementia (MMSE ≤21 or dementia diagnosis) groups.

## Methods

### Participants

Data are from the Newcastle 85+ Study and full details have been published (Collerton *et al.,* 2007; Collerton *et al.,* 2009). In brief, the sampling frame comprised all people born in 1921, aged 85 years in 2006 when recruitment commenced, who were registered with a participating general practice (GP) in Newcastle upon Tyne in the north‐east of England. All individuals who meet these inclusion criteria were invited to participate, whether living at home or in an institution and regardless of their health status (except for exclusion by their general practitioner due to end stage terminal illness). In total, 1459 individuals were invited to participate and 358 (24.5%) declined (Collerton *et al.,* 2009). Recruitment and baseline assessment took place over a 17‐month period beginning in 2006. Of the 1042 people recruited, 845 agreed to a health assessment and a review of their GP records. These individuals form the basis of our analytical sample. Participants were re‐seen at 18 (phase 1), 36 (phase 2) and 60 (phase 3) months.

The Newcastle 85+ Study sample is broadly representative of the local population (Collerton *et al.,* 2009). To investigate whether differential response might affect the results, comparison between the refusers and those agreeing to the health assessment and record review found that the groups did not significantly differ in terms of deprivation but there was underrepresentation of females in those who agreed to participate (Collerton *et al.,* 2009).

### Ethics

The Newcastle and North Tyneside 1 Research Ethics Committee approved the study. Informed written consent was obtained from all participants or their consultee (usually a relative) where capacity to consent was assessed as lacking for example because of severe cognitive impairment or dementia.

### Interview

At baseline, a detailed multi‐dimensional health assessment, including questionnaires, measurements (e.g. blood pressure), functional and cognitive tests, was completed and a fasting blood sample collected. A trained research nurse conducted the health assessment in the participant's usual place of residence. The research nurse team also undertook a review of each participants' GP clinical records. Participants could decline elements of the protocol, and this did not constitute exclusion. At follow‐up, repeat measures of core variables were collected including health, cognition and physical function.

For analysis, we extracted socio‐demographic information including age, sex, years of education and place of residence (institutionalised versus non‐institutionalised). Health status including pre‐existing hypertension, cerebrovascular disease (i.e. stroke or transient ischaemic attack or carotid endarterectomy), diabetes (including medication for diabetes in the absence of diagnosis), ischaemic heart disease (IHD; angina or myocardial infarction or coronary artery bypass grafts or coronary angioplasty or coronary stent), peripheral vascular disease (PVD) and dementia was collected from the review of GP records (Collerton *et al.,* 2009). Disability was assessed based on self‐reported ability to perform instrumental and basic activities of daily living (Kempen *et al.,* 1996; Jagger, *et al.,* 2011). The disability scale was formed by the sum of the number of items where a participant reported difficulty and scores were categorised into four groups: disability free (0), mild (1–6), moderate (7–12) and severe (13+) disability.

### Cognitive measures

Global cognitive function was assessed using the MMSE (Folstein *et al.,* 1975; Molloy and Standish, 1997). MMSE scores range from 0 to 30. The MMSE was administered at baseline, 36 and 60 months. Speed, attention and episodic memory were assessed using the Cognitive Drug Research (CDR) computerised system (Simpson *et al.,* 1991; Ballard *et al.,* 2002; Wesnes *et al.,* 2005; Wesnes, 2008). The CDR was presented on a hi‐resolution Windows‐based tablet computer with a two‐button (yes/no) response box. Administration of the CDR battery was completed in two stages. A training session was performed to familiarise participants with the computerised testing procedures, using all selected tasks but with fewer stimuli. Approximately 1 week later the assessment data were collected. CDR tasks, in order of administration, included word presentation, simple reaction time, digit vigilance task, choice reaction time and word recognition (Simpson *et al.,* 1991). A description of each task is available in Table S1. The scores from these tasks were combined to calculate five composite measures: power of attention (PoA; a measure of focused attention, measured in milliseconds), Continuity of Attention (CoA; a measure of sustained attention or vigilance over time, measured as a trade‐off between correct responses and false alarms), response variability (ResV; a measure of fluctuations in attention, measured as a coefficient of variation) and the word recognition accuracy sensitivity index score (a measure of memory, calculated from the formula presented by Frey and Colliver (Frey and Colliver, 1973) that combines the ability to recognise target stimuli with the ability to correctly reject distractors; range −1 to 1). Reaction times (milliseconds) for each of the three attention tasks (simple reaction time, choice reaction time and digit vigilance reaction time) and word recognition speed were also selected for analysis. All reaction time scores were converted to seconds for analysis. The CDR battery was administered at baseline, 18 and 36 months.

For all attention (except CoA) and the speed measures, lower scores indicate better performance. For the MMSE and the memory task (sensitivity index), higher scores indicate better performance.

### Analysis

Using baseline MMSE scores, individuals were categorised into not impaired (26–30), mildly impaired (22–25) or severely impaired (MMSE ≤ 21) groups. All individuals with a diagnosis of dementia at baseline (*n* = 74, 8.8%) were also grouped into the severely impaired category. Group differences in baseline demographic, lifestyle, health and cognitive variables were compared using the *χ* ^2^ statistic for categorical variables or analysis of variance (or Kruskal–Wallis test for non‐normally distributed variables) for continuous variables.

Trajectories of cognitive change were modelled using repeated‐measures mixed‐effects linear models independently for each cognitive group. Because there were only up to three measures per person for each cognitive test, a model describing change as occurring at a constant rate over the entire follow‐up time was fitted. Mixed models were estimated using maximum likelihood estimation, and estimates were robust under a missing at random assumption. All models were adjusted for sex (0 = male, 1 = female), years of education (centred at 7 years, the average number of years of education in the sample) and age at study entry (centred at age 85; this was performed because of small age differences at baseline with age ranging from 84.5–86.6 years, as recruitment took place over a 17‐month period). Given that cerebrovascular disease status differed across the groups, a sensitivity analysis was undertaken by including this variable as a covariate in the models. The pattern of associations was unchanged (data not shown). Analyses were completed using stata version 14.

## Results

### Demographic and baseline cognitive test scores

Of the 845 participants included in the analysis, most (69.8%, *n =* 590) were classified as not cognitively impaired, while 14.8% (*n =* 125) and 15.4% (*n =* 130) were classified as mildly and severely impaired, respectively. Table 1 shows the baseline characteristics, health status and distribution of the baseline MMSE and CDR scores for each group. There were no significant differences in age at baseline, gender, educational attainment and prevalence of hypertension, diabetes, PVD or IHD by cognitive group. In contrast, the severely impaired group had a higher rate of institutionalisation, greater level of disability, higher prevalence of cerebrovascular disease and poorer baseline performance on all cognitive (i.e. MMSE and CDR) measures.

**Table 1:** Sample demographics and baseline cognitive test scores by cognitive group

	Not impaired (*N =* 590)		Mildly impaired (*N =* 125)		Severely impaired (*N =* 130)		*p*‐value^a^
Demographics							
Baseline age mean (SD)	85.5	(0.4)	85.4	(0.4)	85.5	(0.5)	0.214^b^
Female % (*n*)	61.9	(365)	57.6	(72)	68.5	(89)	0.190
Education mean (SD), years	10.0	(1.9)	9.7	(1.7)	9.8	(1.9)	0.199^b^
Institutionalised % (*n*)	0.9	(5)	11.2	(14)	51.4	(67)	<0.001
Health							
Hypertension % (*n*)	59.3	(350)	56.0	(70)	49.2	(64)	0.104
Peripheral vascular disease % (*n*)	6.8	(40)	8.0	(10)	6.9	(9)	0.888
Ischaemic heart disease % (*n*)	32.4	(191)	39.2	(49)	29.2	(38)	0.211
Cerebrovascular disease % (*n*)	17.8	(105)	28.0	(35)	29.2	(38)	0.002
Diabetes % (*n*)	13.2	(78)	12.8	(16)	13.9	(18)	0.969
Dementia % (*n*)	N/A			N/A	56.9	(74)	N/A
Disability score							
0 (disability free) % (*n*)	24.6	(145)	12.8	(16)	2.3	(3)	<0.001
1–6% (*n*)	56.4	(333)	47.2	(59)	28.5	(37)	
7–12% (*n*)	15.3	(90)	26.4	(33)	33.1	(43)	
13 or more % (*n*)	3.7	(22)	13.6	(17)	36.2	(47)	
Median cognitive test scores (IQR)^c^							
Mini‐Mental State Examination	29	(27, 29)	24	(23, 25)	19	(11, 21)	<0.001
Memory: sensitivity index	0.6	(0.5, 0.7)	0.5	(0.3, 0.7)	0.3	(0.1, 0.5)	<0.001
Power of attention, s	1.5	(1.3, 1.6)	1.7	(1.5, 1.8)	1.9	(1.6, 2.3)	<0.001
Continuity of attention, #	55	(52, 57)	52	(45, 57)	46	(33, 53)	<0.001
Response variability	58.1	(50.7, 67.7)	64.0	(56.7, 78.9)	71.0	(59.5, 99.3)	<0.001
Simple reaction time, s	0.4	(0.3, 0.4)	0.5	(0.4, 0.6)	0.5	(0.4, 0.8)	<0.001
Choice reaction time, s	0.6	(0.5, 0.6)	0.6	(0.6, 0.7)	0.8	(0.6, 1.0)	<0.001
Digit vigilance reaction time, s	0.5	(0.5, 0.5)	0.5	(0.5, 0.6)	0.6	(0.5, 0.6)	<0.001
Word recognition speed, s	1.3	(1.0, 1.6)	1.6	(1.2, 2.1)	2.3	(1.5, 3.5)	<0.001

Key # number; IQR, interquartile range; s, seconds; SD, standard deviation.

a Chi‐squared, unless stated otherwise.

b Analysis of variables (ANOVA).

c Differences between groups tested using the Kruskal‐Wallis test.

### Missing

Table 2 shows the pattern of missing data for each cognitive score for the total sample and by cognitive group. The majority of loss to follow‐up occurred between baseline and the first follow‐up interview. Loss to follow‐up over time was greatest in the severely impaired group. When comparing individuals with only bassline MMSE scores (*n =* 361) to those with one or more MMSE follow‐up assessments (*n =* 478), we found no differences in baseline age (*p* = 0.67), gender (*p* = 0.42) or disease status including hypertension (*p* = 0.96), IHD (30.33 vs 36.84, *p* = 0.05) and diabetes (*p* = 0.44) between the two groups. However, those who were seen at follow‐up had higher educational attainment (mean = 10.06 (SD = 1.98) versus mean = 9.72 (SD = 1.67), *p* = 0.008) and baseline MMSE scores (mean = 27.13 (SD = 3.25) versus mean = 24.17 (SD = 6.88), *p* < 0.001), were less likely to be living in an institution (3.14% vs 18.3%, *p* < 0.001) and have PVD (5.23% vs 9.42%, *p* = 0.019), cerebrovascular disease (18.00 vs 24.93, *p =* 0.020), dementia (3.77 vs 13.85, *p* < 0.001) and disability (severe disability 3.77% vs 18.01%, *p* < 0.001), compared with the group seen only at baseline.

**Table 2:** Number of people (%) with data on each cognitive variable (MMSE and CDR scores) at each follow‐up (FU) interview for the total sample and stratified by cognitive group

	Total sample			Not impaired			Mildly impaired			Severely impaired		
	T0	FU1*	FU2**	T0	FU1*	FU2**	T0	FU1*	FU2**	T0	FU1*	FU2**
MMSE	839	470 (56.0)	331 (70.4)	590	369 (62.5)	273 (74.0)	125	67 (53.6)	43 (64.2)	124	34 (27.4)	15 (44.1)
Memory	753	563 (74.8)	412 (73.2)	568	447 (78.7)	344 (77.0)	103	73 (70.9)	51 (69.9)	82	43 (52.4)	17 (39.5)
PoA	753	562 (74.6)	415 (73.8)	567	447 (78.8)	346 (77.4)	104	72 (69.2)	52 (72.2)	82	43 (52.4)	17 (39.5)
CoA	753	562 (74.6)	415 (73.8)	568	447 (78.7)	346 (77.4)	103	72 (69.9)	52 (72.2)	82	43 (52.4)	17 (39.5)
ResV	752	562 (74.7)	413 (73.5)	567	447 (78.8)	345 (77.2)	104	72 (69.2)	52 (72.2)	81	43 (53.1)	16 (37.2)
SRT	761	570 (74.9)	416 (73.0)	569	452 (79.4)	347 (76.8)	104	74 (71.2)	52 (70.3)	88	44 (50.0)	17 (38.6)
CRT	757	567 (74.9)	415 (73.2)	568	450 (79.2)	346 (76.9)	104	73 (41.3)	52 (71.2)	85	44 (51.8)	17 (38.6)
DVRT	756	563 (74.5)	416 (73.9)	568	447 (78.7)	347 (77.6)	104	73 (70.2)	52 (71.2)	84	43 (51.2)	17 (39.5)
WRS	753	563 (74.8)	412 (73.2)	568	447 (78.7)	344 (77.0)	103	73 (70.9)	51 (69.9)	82	43 (52.4)	17 (39.5)

Key: T0, time 0 (baseline, phase 1); CoA, continuity of attention; CRT, choice reaction time; DVRT, digit vigilance reaction time; MMSE, Mini‐Mental State Examination; PoA, power of attention; ResV, response variability; SRT, simple reaction time; WRS, word recognition speed.

The CDR and MMSE were administered at different time points such that

* FU1 = follow‐up 1 (phase 3 for MMSE and phase 2 for CDR measures) and

** FU2 = follow‐up 2 (phase 4 for MMSE and phase 3 for CDR measures).

### Longitudinal changes in cognitive function

**Figure 1:**
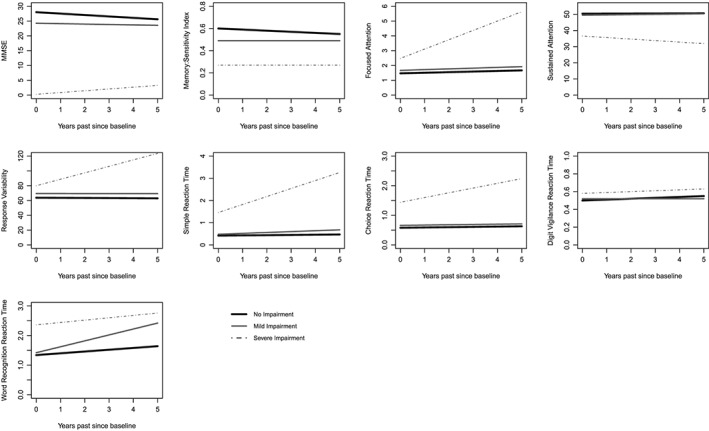
Pattern of changes in MMSE and CDR (memory, attention and speed) scores for each cognitive group over the 5‐year follow‐up.

Table S2 shows the results from the mixed‐effects linear regression analyses for measures of global cognitive function, memory, attention and speed when controlling for age, sex and years of education. Figure 1 shows the trajectories of test scores on each measure for the three cognitive groups over the 5‐years follow‐up.

### Global cognitive function

Over time, there was a significant decline in MMSE scores in the not impaired group (*B* = −0.48, *SE* = 0.15, *p* < 0.005); age, sex and education were not risk factors for the rate of change. In the mildly impaired group, MMSE scores did not change over time. In contrast, in the severely impaired group, higher baseline age was associated with poorer performance over time (*B* = −1.67; *SE* = 0.8, *p* = 0.035).

### Memory: sensitivity index

Memory changed significantly only in the severely impaired group where higher baseline age was associated with poorer performance over time (*B* = −0.10; *SE* = 0.05, *p* = 0.042).

### Attention

In both the not impaired and mildly impaired groups, focused attention (i.e. PoA) scores did not change over time. In contrast, in the severely impaired group, performance declined significantly over time (*B* = 0.63; *SE* = 0.23, *p* = 0.005) and the rate of change differed by sex with slower rates of decline in women (*B* = −0.41; *SE* = 0.19, *p* = 0.033). Sustained attention (i.e. CoA) and response variability (i.e. ResV) scores remained stable over time in all groups.

### Speed: reaction time scores

Results varied across the different speed measures. For simple reaction time scores, only the severely impaired group showed a significant slowing in performance over time (*B* = 0.36 *SE* = 0.14, *p* = 0.012). Choice reaction time scores remained stable over time in all groups. For digit vigilance reaction time, performance declined significantly over time in the not impaired group (*B* = 0.01; *SE* = 0.00, *p* < 0.001), and in the severely impaired group, higher baseline age was associated with a significant decline in performance over time (*B* = 0.04; *SE* = 0.02, *p* = 0.038).

## Discussion

In this study, we examined the trajectories of cognitive performance on measures of global cognitive function, memory, attention and speed in different groups of very‐old individuals defined by MMSE scores and dementia status. The results highlight that groups who were not impaired or mildly impaired at age 85 years had relatively stable performance across most measures, even into their 90th year of life. In contrast, we found decline in the severely impaired group (with the exception of MMSE and digit vigilance reaction times scores that declined significantly in the not impaired group) but not on all measures. Further, in the severely impaired group, very small differences in baseline age appeared to result in poorer performance over time on measures of global cognitive function, memory and speed (digit vigilance task only). Education did not appear to significantly impact the rate of change in any group, and gender differences were rarely observed. The results have implications for the development and targeting of cognitive‐focused intervention strategies for very‐old individuals including strategies aimed at maintaining cognitive integrity in high functioning groups and improving cognition, particularly in the domains of speed and attention, in already impaired individuals. They also highlight that cognitive decline is not an inevitable consequence of advanced ageing and that many very‐old individuals can maintain a high level of cognitive function.

### Cognitive change between ages 85 and 90 years

#### Not and mildly impaired groups

In the not impaired group, global cognitive function declined over time (less than half a point change on the MMSE per year) and digit vigilance speed became slower. In contrast, in the mildly impaired group, cognitive performance remained stable on all measures. Taken together, these results suggest that in the very‐old population, defining not impaired or mildly impaired groups according to MMSE cut‐off scores captures individuals with relatively stable or only slowly changing cognitive function over time. Impairment captured in the mildly impaired group in this population therefore appears to be associated with non‐pathological changes given that there is little risk of decline even into advanced older age.

#### Severely impaired group

In the severely impaired group, while global cognitive function, sustained attention and speed (choice reaction time) remained stable over the 5‐year follow‐up, focused attention declined and speed on the simple reaction time task became slower. Covariates influenced longitudinal performance with higher baseline age associated with poorer global cognitive function, memory and reaction time on the digit vigilance task and being male a risk factor for poorer focused attention. Taken together, these findings suggest that the CDR measures of sustained attention and a simple speed task, rather than memory or the MMSE, are sensitive to capturing change in individuals with severe cognitive impairment/dementia. This supports findings of the usefulness of the CDR in tracking cognitive change in clinical samples with dementia (McKeith *et al.,* 2000; Emre *et al.,* 2004; Wesnes *et al.,* 2010) and extends them to individuals aged 85+ years in a population setting. Future work will determine why some domains and not others show decline over time in the severely impaired group including an in‐depth investigation of the association between cognition and health, lifestyle and reserve factors.

Surprisingly, education was not found to be associated with rate of change on any cognitive measure across the different groups. This is in line with previous null findings (Piccinin *et al.,* 2013). Generally, in longitudinal studies, education is found to be associated with the intercept, not the slope, as reported here. In contrast, in cross‐sectional studies and for dementia risk, education is typically found to be protective. Indeed, in both the 90+ Study and WISE, lower educational attainment was found to be associated with an increased risk of dementia in the very‐old. Therefore, further work exploring reasons behind the results and investigating the link between educational attainment and other proxies of reserve (i.e. occupational level and engagement in mentally stimulating activities) and risk of dementia in the Newcastle 85+ Study is needed to better determine the role of education on cognitive function in this age group.

#### Strengths and limitations

There are a number of strengths to this study. The Newcastle 85+ Study is a large population‐based cohort of the very‐old, socio‐demographically representative of this age group in the UK, including individuals in institutions (Collerton *et al.,* 2009). Home‐based assessments were undertaken that helps to avoid selection bias inherent in clinic‐based assessment of this age group. In addition, relatively long follow‐up has been undertaken (5 years) with little loss to follow‐up apart from death (Davies *et al.,* 2010). The cognitive battery incorporated a wide variety of tests including measures of global and domain specific cognitive function: memory, attention and speed. There are some limitations. First, not all cognitive measures were administered at every follow‐up wave because of time restrictions. Second, as with any study of ageing, follow‐up is associated with loss, mainly due to death, and when looking at attrition rates across groups, this was especially evident in the severely impaired group. Further, loss to follow‐up was found to be associated with lower educational attainment, poor functional, cognitive and health status. This differential loss could affect generalisability of the results. However, we used mixed‐effects regression that controls for time in addition to bias resulting from incomplete observations. Last, given that each cognitive measure (MMSE and CDR) was administered at three time points, only linear change could be modelled.

## Conclusion

In the very‐old, cognitive function generally remained stable over time in not impaired and mildly impaired groups but did decline significantly, especially in measures of attention and speed, once a threshold is met (e.g. an individual is severely impaired/has dementia). Education was not protective against rate of decline. Poorer performance was associated with increasing age for some but not all measures at follow‐up in the severely impaired group, whilst gender differences were not consistently observed. Further research is needed to identify risk factors for cognitive decline in the different cognitive groups and to develop strategies to improve cognitive function or prevent further impairment in individuals with severe impairments. This is particularly important as lifespan continues to increase and the ageing population expands.

## Ethics Statement

Initial approval was given by Newcastle and North Tyneside Local Research Ethics Committee 1 on 24 February 2006. The REC reference number, The Newcastle 85+ Study: Biological, Clinical and Psychosocial factors associated with healthy ageing, is 06/Q0905/2. Written informed consent was obtained from participants; where individuals lacked capacity to consent, for example, because of cognitive impairment, a formal written opinion was sought from a relative or carer.

## Conflict of interest

Dr Saxby is an ex‐employee of Cognitive Drug Research (CDR) Ltd, a commercial provider of the CDR computerised system, now owned by Bracket Global, Goring‐on‐Thames, UK, which provides the CDR system to the clinical trials industry. Dr Wesnes, the inventor of the CDR system, is an ex‐employee and stockholder of Bracket Global now employed by Wesnes Cognition Ltd, Streatley‐on‐Thames, UK. At the time of the Newcastle 85+ Study, the CDR was a proprietary system owned by Dr Wesnes, who provided it to the study on a non‐commercial basis.

## Author contributions

Conceived and designed the experiments: BCM, GM, AG, JC, KD, BKS, KW, TBLK and CJ. Analysed the data: BCMS and GM. Writing and revising of the manuscript: BCM, GM, AG, JC, KD, BKS, KW, TBLK and CJ. Approved the final manuscript: BCM, GM, AG, JC, KD, BKS, KW, TBLK and CJ.

**Key points**

- In the very‐old (i.e. persons ≥85 years), cognitive function remains relatively stable over time in not impaired and mild cognitively impaired groups, but declines, especially on measures of attention and speed, in individuals with severe impairment/dementia.
- Cognitive decline is not an inevitable consequence of advanced ageing; many very‐old individuals can maintain high cognitive function into their 90th year of life.
- Determining factors that promote successful cognitive functioning in advanced old age will have important implications for developing strategies to prevent cognitive decline in this age group.

## Supporting information


**Table S1** Cognitive Drug Research (CDR) Assessment Battery tasks and outcome scores used in the Newcastle 85+ Study
**Table S2** Results of the mixed multilevel analyses for the MMSE and CDR memory, attention and speed scores for each cognitive group controlling for age, sex and years of education
