# Supplementary material for: Longitudinal changes in global and domain specific cognitive function in the very‐old: findings from the Newcastle 85+ Study
Source: Int J Geriatr Psychiatry. 2017 Jun 22;33(2):298–306. doi: 10.1002/gps.4743 (PMC5811803; doi:10.1002/gps.4743)
Supplement: Supplementary file 1 — Table S1 Cognitive Drug Research (CDR) Assessment Battery tasks and outcome scores used in the Newcastle 85+ Study Table S2 Results of the mixed multilevel analyses for the MMSE and CDR memory, attention and speed scores for each cognitive group controlling for age, sex and years of education [file GPS-33-298-s001.docx]

**Online Supplementary Table 1** Cognitive Drug Research (CDR) Assessment Battery tasks and outcome scores used in the Newcastle 85+ Study

| CDR Task | Description | Outcome Variables |
| --- | --- | --- |
| Word Presentation | A list of 15 words are presented on the screen at a rate of 1 word every 2 seconds for the participant to read and remember | No data recorded for this task |
| Simple Reaction Time | The participant is instructed to press “YES” as quickly as possible every time the word “YES” is presented on the screen. In total, 30 “YES” stimuli are presented with varying inter-stimulus interval | Mean reaction time (milliseconds, ms) |
| Choice Reaction Time | Either the word “YES” or “NO” is presented on the screen and the participant is instructed to press the corresponding button as quickly as possible. There are 30 trials for each stimulus word, which is chosen randomly with equal probability, with varying inter-stimulus interval | Mean reaction time (ms)  Choice RT accuracy (# errors) |
| Digit Vigilance Task | A target digit is randomly selected and constantly displayed to the right of the screen. A series of digits (0-9) are presented in the centre of the screen at the rate of 150 per minute. The participant is required to press the “YES” button as quickly as possible every time the digit in the series matches the target digit. There are 300 digits in the series and the task lasts for 2 minutes | Digit Vigilance Speed (mean react time, ms)  Targets Detected (%)  Number of false alarms (# errors) |
| Word Recognition | The original 15 words plus 15 distractor words are presented one at a time in a randomised order. For each word the participant is required to indicate whether or not they recognise it from the original list by pressing the “YES” or “NO” button as appropriate and as quickly as possible. Following the response there is a delay of 1 second before the next word is presented | Accuracy (% of correct responses to target stimuli),  Word Recognition Speed (mean reaction time of correct responses, ms)  Sensitivity Index (SI)* |

**Notes**

*A measure of accuracy combining the ability to recognise target stimuli with the ability to correctly reject distractors. A sensitivity index of 1 represents perfect discrimination between correct and distractor items and a score of 0 chance performance. Negative sensitivity scores can be obtained with poor performance that is worse than chance.

**Online Supplementary Table 2** Results of the mixed multilevel analyses for the MMSE and CDR memory, attention and speed scores for each cognitive group controlling for age, sex and years of education

|  | **Not Impaired** | | | **Mildly Impaired** | | | **Severely Impaired** | | |
| --- | --- | --- | --- | --- | --- | --- | --- | --- | --- |
|  | **Coef.** | **(Std. Err.)** | **p-value** | **Coef.** | **(Std. Err.)** | **p-value** | **Coef.** | **(Std. Err.)** | **p-value** |
| **MMSE** |  |  |  |  |  |  |  |  |  |
| **INTERCEPT** |  |  |  |  |  |  |  |  |  |
| Constant | 28.00 | (0.15) | 0.000 | 24.26 | (0.24) | 0.000 | 15.06 | (1.52) | 0.000 |
| Age | **0.45** | **(0.15)** | **0.002** | 0.18 | (0.27) | 0.497 | **3.54** | **(1.49)** | **0.018** |
| Sex | **0.27** | **(0.13)** | **0.035** | -0.10 | (0.21) | 0.628 | 0.37 | (1.44) | 0.798 |
| Education | **0.09** | **(0.03)** | **0.003** | -0.05 | (0.07) | 0.473 | 0.33 | (0.34) | 0.339 |
| **SLOPE** |  |  |  |  |  |  |  |  |  |
| Rate of change per year | **-0.48** | **(0.15)** | **0.002** | -0.14 | (0.40) | 0.722 | 0.60 | (0.85) | 0.479 |
| Age | -0.14 | (0.13) | 0.262 | -0.52 | (0.37) | 0.167 | **-1.67** | **(0.79)** | **0.035** |
| Sex | 0.02 | (0.12) | 0.835 | -0.49 | (0.33) | 0.143 | -1.19 | (0.74) | 0.108 |
| Education | 0.01 | (0.03) | 0.832 | 0.05 | (0.10) | 0.610 | -0.08 | (0.17) | 0.657 |
| **Memory: Sensitivity Index** |  |  |  |  |  |  |  |  |  |
| **INTERCEPT** |  |  |  |  |  |  |  |  |  |
| Constant | 0.60 | (0.02) | 0.000 | 0.49 | (0.06) | 0.000 | 0.27 | (0.09) | 0.002 |
| Age | 0.02 | (0.02) | 0.460 | 0.06 | (0.06) | 0.287 | 0.10 | (0.09) | 0.273 |
| Sex | -0.02 | (0.02) | 0.348 | -0.07 | (0.05) | 0.151 | -0.08 | (0.08) | 0.319 |
| Education | 0.01 | (0.00) | 0.301 | 0.00 | (0.02) | 0.916 | 0.01 | (0.02) | 0.604 |
| **SLOPE** |  |  |  |  |  |  |  |  |  |
| Rate of change per year | -0.01 | (0.01) | 0.254 | 0.00 | (0.02) | 0.883 | 0.00 | (0.05) | 0.987 |
| Age | -0.01 | (0.01) | 0.340 | -0.02 | (0.02) | 0.321 | **-0.10** | **(0.05)** | **0.042** |
| Sex | 0.00 | (0.01) | 0.878 | -0.01 | (0.02) | 0.763 | 0.02 | (0.04) | 0.575 |
| Education | 0.00 | (0.00) | 0.688 | -0.01 | (0.01) | 0.228 | -0.01 | (0.01) | 0.453 |
| **Focused Attention: Power of Attention** |  |  |  |  |  |  |  |  |  |
| **INTERCEPT** |  |  |  |  |  |  |  |  |  |
| Constant | 1.47 | (0.03) | 0.000 | 1.67 | (0.15) | 0.000 | 2.48 | (0.41) | 0.000 |
| Age | **-0.07** | **(0.03)** | **0.021** | 0.02 | (0.15) | 0.887 | -0.61 | (0.42) | 0.150 |
| Sex | **0.11** | **(0.03)** | **0.000** | 0.09 | (0.12) | 0.451 | 0.38 | (0.37) | 0.306 |
| Education | -0.01 | (0.01) | 0.117 | 0.00 | (0.04) | 0.972 | -0.08 | (0.08) | 0.329 |
| **SLOPE** |  |  |  |  |  |  |  |  |  |
| Rate of change per year | 0.04 | (0.02) | 0.074 | 0.05 | (0.06) | 0.420 | **0.63** | **(0.23)** | **0.005** |
| Age | 0.01 | (0.02) | 0.702 | 0.02 | (0.06) | 0.782 | -0.25 | (0.23) | 0.285 |
| Sex | 0.01 | (0.02) | 0.642 | 0.00 | (0.05) | 0.951 | **-0.41** | **(0.19)** | **0.033** |
| Education | 0.00 | (0.00) | 0.364 | 0.01 | (0.02) | 0.531 | 0.01 | (0.04) | 0.844 |
| **Sustained Attention: Continuity of Attention** |  |  |  |  |  |  |  |  |  |
| **INTERCEPT** |  |  |  |  |  |  |  |  |  |
| Constant | 50.40 | (0.68) | 0.000 | 49.48 | (2.27) | 0.000 | 36.57 | (3.65) | 0.000 |
| Age | **1.43** | **(0.63)** | **0.023** | -4.01 | (2.29) | 0.080 | **7.75** | **(4.01)** | **0.053** |
| Sex | **2.28** | **(0.56)** | **0.000** | 2.54 | (1.89) | 0.179 | 3.74 | (3.46) | 0.280 |
| Education | **0.48** | **(0.14)** | **0.001** | 0.23 | (0.64) | 0.722 | 0.04 | (0.78) | 0.960 |
| **SLOPE** |  |  |  |  |  |  |  |  |  |
| Rate of change per year | 0.04 | (0.32) | 0.897 | 0.17 | (0.88) | 0.847 | -0.94 | (2.19) | 0.668 |
| Age | -0.38 | (0.27) | 0.165 | 0.59 | (0.88) | 0.503 | -0.30 | (2.24) | 0.892 |
| Sex | -0.07 | (0.25) | 0.790 | -1.14 | (0.78) | 0.142 | -0.14 | (1.9) | 0.940 |
| Education | -0.11 | (0.06) | 0.078 | -0.22 | (0.24) | 0.367 | -0.43 | (0.39) | 0.274 |
| **Fluctuations in Attention: Response Variability** |  |  |  |  |  |  |  |  |  |
| **INTERCEPT** |  |  |  |  |  |  |  |  |  |
| Constant | 63.76 | (1.61) | 0.000 | 69.38 | (5.8) | 0.000 | 79.96 | (10.61) | 0.000 |
| Age | -2.90 | (1.52) | 0.057 | 2.87 | (5.78) | 0.620 | 11.14 | (11.37) | 0.327 |
| Sex | 0.30 | (1.35) | 0.827 | 1.26 | (4.83) | 0.794 | -4.85 | (9.87) | 0.623 |
| Education | **-0.94** | **(0.33)** | **0.004** | -0.51 | (1.61) | 0.754 | 0.43 | (2.13) | 0.838 |
| **SLOPE** |  |  |  |  |  |  |  |  |  |
| Rate of change per year | -0.16 | (0.89) | 0.857 | -0.03 | (1.71) | 0.987 | 8.73 | (6.84) | 0.202 |
| Age | 1.04 | (0.77) | 0.175 | 0.91 | (1.73) | 0.598 | -4.30 | (6.81) | 0.527 |
| Sex | -0.57 | (0.70) | 0.415 | -0.92 | (1.53) | 0.548 | -2.38 | (5.94) | 0.688 |
| Education | 0.25 | (0.17) | 0.139 | 0.18 | (0.47) | 0.695 | -0.14 | (1.22) | 0.909 |
| **Simple Reaction Time** |  |  |  |  |  |  |  |  |  |
| **INTERCEPT** |  |  |  |  |  |  |  |  |  |
| Constant | 0.42 | (0.02) | 0.000 | 0.48 | (0.06) | 0.000 | 1.46 | (0.34) | 0.000 |
| Age | **-0.05** | **(0.02)** | **0.002** | 0.02 | (0.07) | 0.756 | -0.30 | (0.34) | 0.367 |
| Sex | **0.03** | **(0.01)** | **0.026** | 0.02 | (0.05) | 0.772 | -0.04 | (0.31) | 0.895 |
| Education | -0.01 | (0) | 0.088 | 0.00 | (0.02) | 0.840 | -0.13 | (0.07) | 0.066 |
| **SLOPE** |  |  |  |  |  |  |  |  |  |
| Rate of change per year | 0.01 | (0.01) | 0.337 | 0.04 | (0.04) | 0.293 | **0.36** | **(0.14)** | **0.012** |
| Age | 0.01 | (0.01) | 0.241 | -0.01 | (0.04) | 0.873 | -0.24 | (0.15) | 0.104 |
| Sex | 0.01 | (0.01) | 0.238 | -0.01 | (0.04) | 0.695 | -0.14 | (0.12) | 0.239 |
| Education | 0.00 | (0.00) | 0.590 | 0.00 | (0.01) | 0.761 | 0.00 | (0.03) | 0.866 |
| **Choice Reaction Time** |  |  |  |  |  |  |  |  |  |
| **INTERCEPT** |  |  |  |  |  |  |  |  |  |
| Constant | 0.58 | (0.02) | 0.000 | 0.66 | (0.07) | 0.000 | 1.44 | (0.23) | 0.000 |
| Age | -0.03 | (0.02) | 0.056 | 0.01 | (0.07) | 0.922 | -0.37 | (0.25) | 0.138 |
| Sex | **0.05** | **(0.01)** | **0.002** | 0.06 | (0.06) | 0.305 | -0.14 | (0.22) | 0.507 |
| Education | -0.01 | (0) | 0.071 | -0.01 | (0.02) | 0.745 | -0.05 | (0.05) | 0.269 |
| **SLOPE** |  |  |  |  |  |  |  |  |  |
| Rate of change per year | 0.01 | (0.01) | 0.366 | 0.01 | (0.03) | 0.728 | 0.16 | (0.1) | 0.120 |
| Age | 0.00 | (0.01) | 0.802 | 0.03 | (0.03) | 0.373 | 0.00 | (0.11) | 0.997 |
| Sex | 0.00 | (0.01) | 0.625 | 0.01 | (0.03) | 0.832 | -0.12 | (0.09) | 0.172 |
| Education | 0.00 | (0) | 0.178 | 0.01 | (0.01) | 0.345 | 0.01 | (0.02) | 0.743 |
| **Digit Vigilance Reaction Time** | |  |  |  |  |  |  |  |  |
| **INTERCEPT** |  |  |  |  |  |  |  |  |  |
| Constant | 0.50 | (0.01) | 0.000 | 0.52 | (0.02) | 0.000 | 0.58 | (0.03) | 0.000 |
| Age | -0.01 | (0.01) | 0.314 | 0.00 | (0.02) | 0.810 | **-0.07** | **(0.03)** | **0.012** |
| Sex | **0.02** | **(0.01)** | **0.000** | 0.03 | (0.02) | 0.102 | **0.05** | **(0.02)** | **0.031** |
| Education | 0.00 | (0.00) | 0.951 | 0.01 | (0.01) | 0.239 | -0.01 | (0.01) | 0.316 |
| **SLOPE** |  |  |  |  |  |  |  |  |  |
| Rate of change per year | **0.01** | **(0.00)** | **0.000** | 0.00 | (0.01) | 0.820 | 0.01 | (0.02) | 0.655 |
| Age | 0.00 | (0.00) | 0.665 | 0.00 | (0.01) | 0.638 | **0.04** | **(0.02)** | **0.038** |
| Sex | 0.00 | (0.00) | 0.246 | 0.00 | (0.01) | 0.888 | -0.03 | (0.01) | 0.057 |
| Education | 0.00 | (0.00) | 0.561 | 0.00 | (0.00) | 0.823 | 0.00 | (0.00) | 0.521 |
| **Word Recognition Speed** | |  |  |  |  |  |  |  |  |
| **INTERCEPT** |  |  |  |  |  |  |  |  |  |
| Constant | 1.34 | (0.07) | 0.000 | 1.42 | (0.23) | 0.000 | 2.36 | (0.42) | 0.000 |
| Age | 0.02 | (0.07) | 0.757 | -0.31 | (0.24) | 0.197 | -0.54 | (0.45) | 0.235 |
| Sex | **0.19** | **(0.06)** | **0.001** | **0.54** | **(0.20)** | **0.006** | **0.82** | **(0.39)** | **0.037** |
| Education | -0.02 | (0.02) | 0.159 | 0.05 | (0.07) | 0.416 | -0.10 | (0.09) | 0.259 |
| **SLOPE** |  |  |  |  |  |  |  |  |  |
| Rate of change per year | 0.06 | (0.03) | 0.087 | 0.20 | (0.13) | 0.130 | 0.08 | (0.34) | 0.823 |
| Age | -0.01 | (0.03) | 0.816 | 0.12 | (0.13) | 0.363 | 0.65 | (0.34) | 0.058 |
| Sex | **0.05** | **(0.03)** | **0.044** | -0.01 | (0.12) | 0.937 | -0.43 | (0.29) | 0.141 |
| Education | 0.00 | (0.01) | 0.861 | -0.01 | (0.04) | 0.703 | 0.04 | (0.06) | 0.520 |

**Key**

**Coef.** Beta Coefficient; **SE** Standard Error

**Notes** Significant (p < 0.05) results shown in bold
